# Exploring Urological Malignancies on Pinterest: Content Analysis

**DOI:** 10.2196/36244

**Published:** 2022-08-22

**Authors:** Amber S Herbert, Naeemul Hassan, Rena D Malik, Stacy Loeb, Akya Myrie

**Affiliations:** 1 Stanford School of Medicine Department of Urology Stanford, CA United States; 2 University of Maryland, College Park College of Information Studies College park, MD United States; 3 Division of Urology Department of Surgery University of Maryland Medical Center Baltimore, MD United States; 4 New York University School of Medicine Department of Urology New York, NY United States; 5 Glickman Urological and Kidney Institute Cleveland Clinic Department of Urology Cleveland, OH United States

**Keywords:** bladder cancer, Pinterest, prostate cancer, kidney cancer, testicular cancer, urological cancer, misinformation, genitourinary, malignancy, oncology, content, information, social media, accuracy, quality

## Abstract

**Background:**

Pinterest is a visually oriented social media platform with over 250 million monthly users. Previous studies have found misinformative content on genitourinary malignancies to be broadly disseminated on YouTube; however, no study has assessed the quality of this content on Pinterest.

**Objective:**

Our objective was to evaluate the quality, understandability, and actionability of genitourinary malignancy content on Pinterest.

**Methods:**

We examined 540 Pinterest posts or pins, using the following search terms: “bladder cancer,” “kidney cancer,” “prostate cancer,” and “testicular cancer.” The pins were limited to English language and topic-specific content, resulting in the following exclusions: bladder (n=88), kidney (n=4), prostate (n=79), and testicular cancer (n=10), leaving 359 pins as the final analytic sample. Pinterest pins were classified based on publisher and perceived race or ethnicity. Content was assessed using 2 validated grading systems: DISCERN quality criteria and the Patient Education Materials Assessment Tool. The presence of misinformation was evaluated using a published Likert scale ranging from 1=none to 5=high.

**Results:**

Overall, 359 pins with a total of 8507 repins were evaluated. The primary publisher of genitourinary malignancy pins were health and wellness groups (n=162, 45%). Across all genitourinary malignancy pins with people, only 3% (n=7) were perceived as Black. Additionally, Asian (n=2, 1%) and Latinx (n=1, 0.5%) individuals were underrepresented in all pins. Nearly 75% (n=298) of the pins had moderate- to poor-quality information. Misinformative content was apparent in 4%-26% of all genitourinary cancer pins. Understandability and actionability were poor in 55% (n=198) and 100% (n=359) of the pins, respectively.

**Conclusions:**

On Pinterest, the majority of the urological oncology patient-centric content is of low quality and lacks diversity. This widely used, yet unregulated platform has the ability to influence consumers’ health knowledge and decision-making. Ultimately, this can lead to consumers making suboptimal medical decisions. Moreover, our findings demonstrate underrepresentation across many racial and ethnic groups. Efforts should be made to ensure the dissemination of diverse, high-quality, and accurate health care information to the millions of users on Pinterest and other social media platforms.

## Introduction

Social media has expanded rapidly over the past decade and has become a vital part of our day to day lives [1,2]. Increasingly, it is becoming the initial source for patients in search of supplemental information regarding their disease [3]. Users are drawn to the easy accessibility of health care information. Unknowingly, much of the material they encounter is non–evidence-based, leaving them susceptible to misinformation [4].

Social media platforms like Pinterest, Instagram, Twitter, and TikTok are commonly used among younger populations in search of information [2]. Pinterest is the fourth most popular social media site with over 250 million users per month [1]. It is a visually orientated platform with the ability to quickly disseminate medical information to consumers. Consumers from around the world are using social media platforms to search and exchange health-related information [3]. Previous studies have reported the wide dissemination of misinformative content about urological malignancies on YouTube [4,5]. This is primarily because prior studies on the quality of social media content about urological malignancies have focused on YouTube. Urological malignancies misinformation is a concerning phenomenon that requires further analysis on other commonly used platforms. Little is known about the quality of consumer-centric content about urological malignancies on Pinterest. Our objective was to perform the first comprehensive study assessing the quality of content related to bladder, kidney, prostate, and testicular cancer on Pinterest. We hypothesized that most of the consumer information on urological oncology will be of low quality, with poor understandability and actionability, and lacking racial or ethnic diversity.

## Methods

We reviewed 540 Pinterest pins, using the following search terms: “bladder cancer,” “kidney cancer,” “prostate cancer,” and “testicular cancer” via an application programming interface. Pins were excluded if they did not contain relevant content (ie, if they did not mention gallbladder or thyroid cancer) or if they were not in English. This resulted in the following excluded data: bladder (n=88), kidney (n=4), prostate (n=79), and testicular cancer (n=10). Two reviewers independently scored each pin and linked content. Interrater discrepancies were addressed by group discussion.

Pins were assessed using 2 validated questionnaires: the DISCERN quality criteria and Patient Education Materials Assessment Tool (PEMAT) [6,7]. The DISCERN questionnaire assesses consumer health information using 16 items that are scored from 1 to 5 (ie, no to yes) [6]. PEMAT evaluates the understandability and actionability of patient education resources, using a questionnaire containing 17 items (13 on understandability and 4 on actionability) that are scored as “agree,” “disagree,” or “not applicable” [7]. Misinformation was characterized using a previously published Likert scale, ranging from 1=none to 5=high [5]. We also evaluated the presence of commercial bias (ie, link to paid subscription or endorsement of a service or product). Reviewers further examined the dissemination of information by calculating the number of repins and followers associated with the Pinterest posts. The action of repinning copies the image and adds the image to the user’s Pinterest board [1]. Finally, to examine the diversity of racial or ethnic representation, reviewers classified people in pins based on perceived race and ethnicity, as was done in previous studies [8]. Race was categorized as Black, White, Asian, or unknown (ie, unable to discern). Ethnicity was classified as Latinx, non-Latinx, or unknown (ie, unable to discern).

## Results

### Pin Characteristics

**Figure 1:**
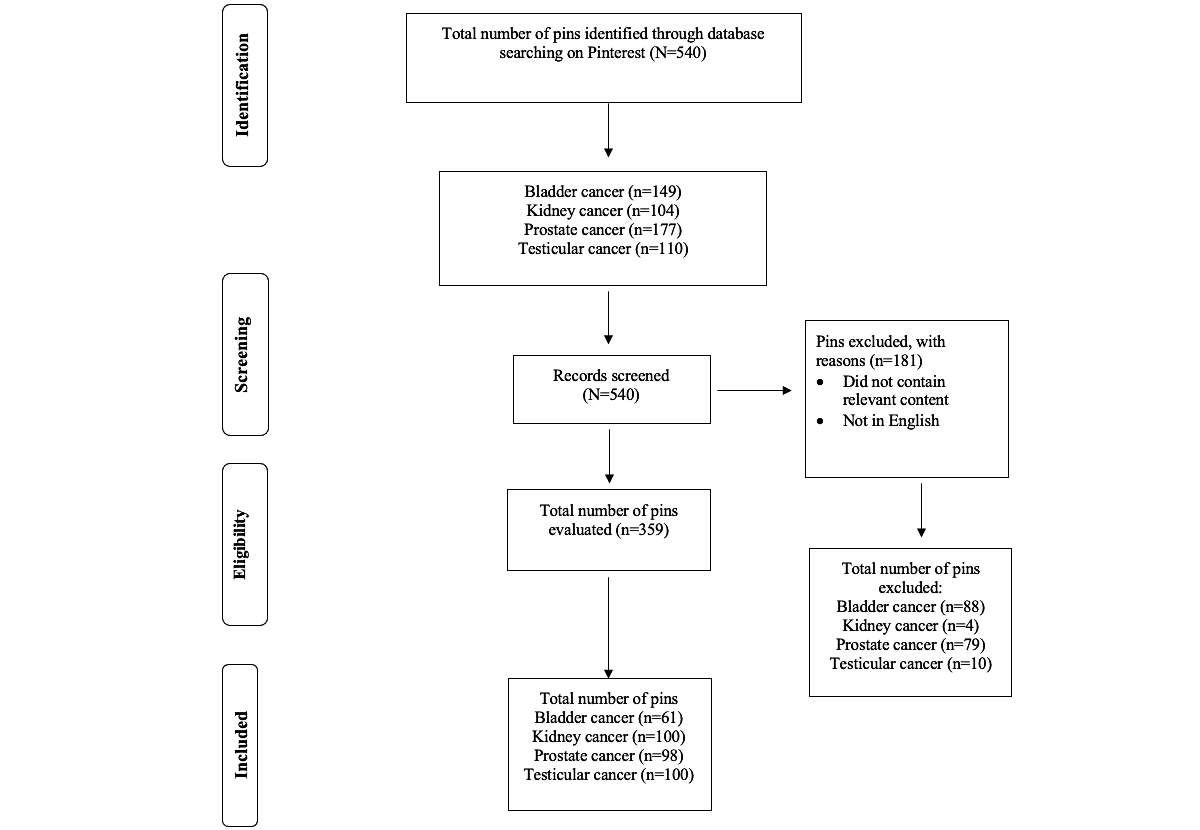
PRISMA (Preferred Reporting Items for Systematic reviews and Meta-Analyses) diagram for urological malignancies on Pinterest (reproduced from Moher et al [9]).

**Figure 2:**
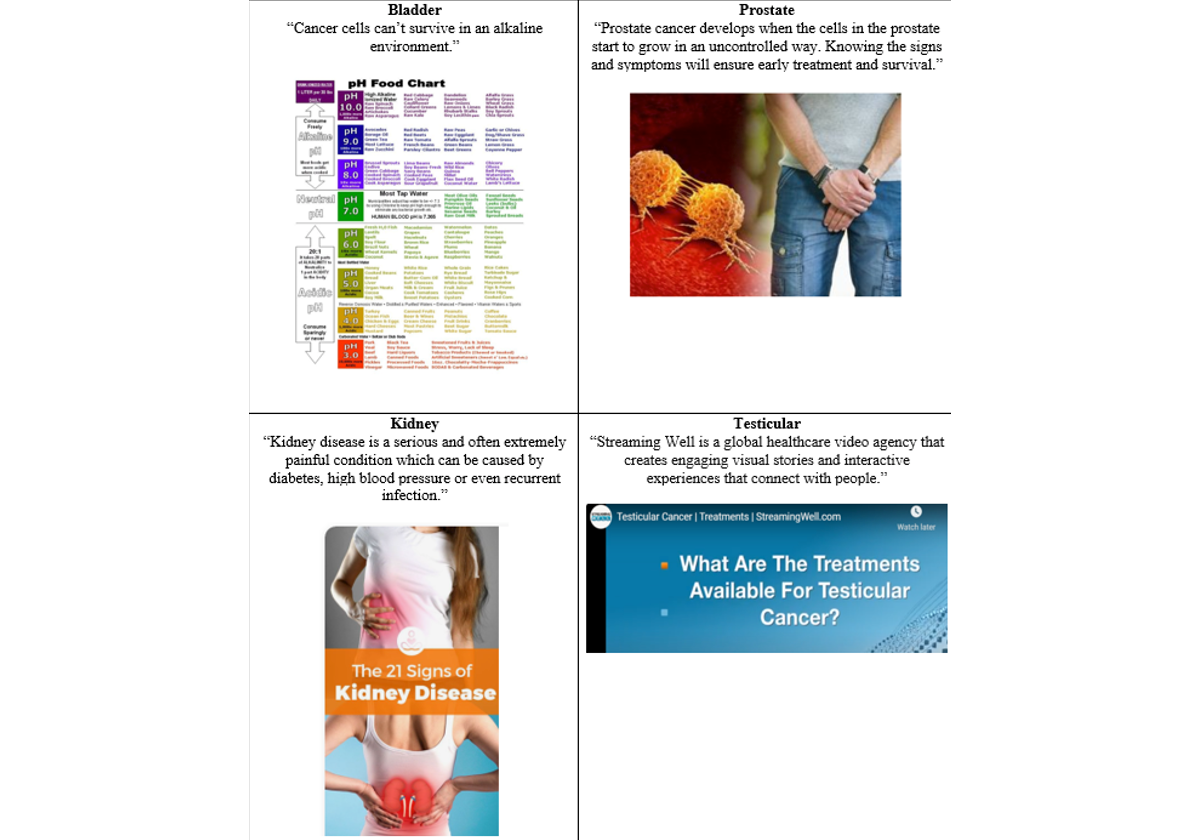
Highest repins for each urological malignancy.

In total, 359 pins met the inclusion criteria (Table 1). The total pins excluded (Figure 1) per topic were the following: bladder (n=88), kidney (n=4), prostate (n=79), and testicular cancer (n=10). On average, bladder, kidney, prostate, and testicular cancer pins had 175,874 followers and 25 repins. The highest repins per topic were for bladder (n=521), kidney (n=1361), prostate (n=40), and testicular cancer (n=15; Figure 2). Testicular cancer had the lowest average number of followers. Bladder cancer and kidney cancer had higher mean repins. The majority of the urological cancer pins were published by health or wellness groups (n=162, 45%), followed by health care–based groups (n=57, 15%), that is, from hospitals or clinics, doctors, academic journals, and medical education.

**Table 1:** Analysis of urological oncology content on Pinterest (N=359).

Characteristics			Urological oncology content						
			Bladder (n=61)		Kidney (n=100)		Prostate (n=98)		Testicular (n=100)
Average number of followers, n			152,591		109,716		364,917		76,273
Average number of repins, n (range)			38 (1-521)		59 (1-1361)		2 (1-40)		1 (1-15)
**Publisher type, n (%)**									
	Health care–based	9 (15)		14 (14)		18 (18)		16 (16)	
	Consumer or patient	5 (8)		10 (10)		2 (2)		3 (3)	
	Foundational or advocacy group	4 (7)		8 (8)		7 (7)		16 (16)	
	Governmental	2 (3)		0 (0)		1 (1)		3 (3)	
	News source or media outlet	1 (2)		6 (6)		11 (11)		5 (5)	
	Commercial media or industry	12 (20)		12 (12)		13 (13)		13 (13)	
	Health and wellness	28 (46)		50 (50)		41 (42)		43 (43)	
	Unknown/other	0 (0)		8 (7.6)		6 (6)		1 (1)	
**Race, n/N (%)^a^**									
	Black	0/43 (0)		2/63 (3)		2/39 (5)		3/63 (5)	
	White	41/43 (95)		54/63 (88)		30/39 (77)		53/63 (84)	
	Asian	0/43 (0)		2/63 (3)		0/39 (0)		0/63 (0)	
	Unknown	2/43 (5)		3/63 (5)		5/39 (13)		7/63 (11)	
**Ethnicity, n/N (%)^a^**									
	Latinx	0/43 (0)		0/63 (0)		0/39 (0)		1/63 (1)	
	Non-Latinx	41/43 (95)		58/63 (92)		32/39 (82)		53/63 (84)	
	Unknown	2/43 (5)		3/63 (5)		7/39 (18)		9/63 (14)	
**Characteristics discussed, n (%)**									
	Anatomy	15 (25)		37 (37)		33 (34)		43 (43)	
	Symptoms	16 (26)		29 (29)		14 (14)		30 (30)	
	Detection	4 (7)		8 (8)		10 (10)		30 (30)	
	Treatment	4 (7)		8 (8)		8 (8)		10 (10)	
	Side effects of treatment	0 (0)		0 (0)		2 (2)		4 (4)	
	Lifestyle or dietary modification	25 (41)		21 (21)		44 (45)		8 (8)	
Commercial bias present, n (%)			4 (7)		1 (1)		14 (14)		0 (0)
Misinformation^b^, n (%)			16 (26)		9 (9)		15 (15)		4 (4)
Shared decision-making, n (%)			1 (1)		2 (2)		3 (3)		5 (5)
Quality score ≤3, n (%)			50 (82)		87 (87)		94 (96)		67 (67)
PEMAT^c^ understandability <75%, n (%)			37 (61)		62 (62)		69 (70)		30 (30)
PEMAT actionability <75%, n (%)			61 (100)		100 (100)		97 (99)		100 (100)

^a^“N” refers to the total number of people depicted in pins and “n” refers to the specific number of people based on perceived race and ethnicity.

^b^Likert score >1 out of 5.

^c^PEMAT: Patient Education Materials Assessment Tool.

### Quality of Pins

The overall quality of pins was low. Nearly three-fourths of the pins contained moderate- to poor-quality information, or a DISCERN ≤3. Poor-quality pins do not state its purpose, have relevant content, identify sources of information, address quality of life, risks of treatment, or other available treatment options. Nearly all pins failed to mention shared decision-making (n=348, 97%). Misinformation ranged from 4% (n=4) in testicular cancer to 26% (n=16) in bladder cancer pins (eg, cow urine for the treatment of bladder cancer). Over 60% (n=198) of bladder, kidney, and prostate cancer pins had low PEMAT scores for understandability, suggesting many of the pins were not easy to understand. Nearly all pins had low PEMAT scores for actionability, indicating they did not have readily actionable information for users.

### Racial and Ethnic Demographics

Among the 206 total people depicted across all pins, the majority were perceived as White (n=178, 86%) and non-Latinx (n=184, 89%). Only 3% (n=7) of people were perceived as Black. Bladder cancer pins did not include a Black individual. Additionally, fewer than 1% (n=2) of individuals represented in pins were perceived as Asian.

## Discussion

### Principal Findings

This is the first study to comprehensively assess the quality of urological oncology content on Pinterest. We found that testicular cancer had fewer followers than other reported urological malignancies. This is not surprising as testicular diseases are less common, only affecting approximately 1% of men [10]. A concerning finding was the spread of misinformation on this platform, with one-fourth of bladder cancer pins containing misinformation, primarily shared through nonhospital and non–peer-reviewed websites. Urological oncology content on Pinterest also lacks actionable information, leaving users perplexed on what their next steps should be. Moreover, there is a paucity of racial and ethnic diversity within the urological oncology content present on Pinterest.

### Comparison With Prior Work

As the intersection between social media and medicine expands, the dissemination of misinformative and inaccurate content on social media platforms is becoming a major societal concern. We found that 26% (n=16) of bladder cancer pins contained misinformation. This aligns with the findings of previous studies that showed 29% of the top YouTube bladder cancer videos had misinformative content [11]. Similarly, a prior study evaluating the quality of breast cancer information on Pinterest found that over half of the pins contained misinformation. Although we do not know the full impact of this content on users’ decision-making capabilities, we are aware that they are frequently shared. Alsyouf et al [12] found that inaccurate or misleading articles on urological cancers were 28 times more likely to be shared on Facebook, Pinterest, Twitter, and Reddit in comparison to fact-based articles. This highlights how patients are susceptible to misinformation and the potential influence it can have on their medical decision-making [12]. Pinterest is primarily used as a search engine, and we hope that medical providers will link useful content to Pinterest to increase the quality of information available to users on urological malignancies [13]. Pinterest, like other social media platforms, is a powerful medium with the ability to enhance the knowledge of lay users; however, it has the propensity to disseminate misinformative content.

Approximately all urological oncology content on Pinterest lacks actionable information. Previous studies evaluating the actionability of prostate cancer information on YouTube found that over two-thirds of videos contained actionable content. We reported 99% (n=358) of the pins lacked actionable content or the ability to determine the next steps of action. This is likely due to the brevity of the pins content, which mostly focused on the symptoms associated with various urological conditions. Despite these findings, prior literature has found that well-informed patients have better health-related outcomes and are better able to identify and seek help for their symptoms [14]. Comprehensive patient educational materials that describe actionable steps may help patients determine urgency in seeking medical care [14]. Ultimately, generating patient-centric information that enhances the ability to comprehend their disease will improve shared decision-making among patients and providers [15].

This study corroborates the paucity of racial or ethnic representation of urological malignances on social media [8]. Borno et al [8] found that only 4% of people depicted in YouTube videos on prostate cancer were perceived as Black. African Americans are disproportionately affected by certain urological cancers (ie, prostate cancer) and should have a better representation in patient-centric educational content. Nearly half of Black individuals screened reported receiving health care information from web-based sources [16]. We must ensure that accurate and reliable information is disseminated to make more informed decisions. Across social media platforms, there is a critical need for diverse, actionable, and high-quality patient education materials to help improve health outcomes.

### Limitations

Our study is limited to Pinterest, which is just one of many web-based networks. However, since Pinterest is the fourth most commonly used social media platform and no study to date has assessed its urological oncology content, our results fill an important gap. Also, the application of the validated questionaries to the Pinterest interface is a limitation. More work is needed to further develop methods in quality assessment across different social media platforms [17]. We are limited to the subjective nature of pin scoring among reviewers. Efforts were made to mitigate this through the use of validated instruments to assess consumer health information and perform coding comparisons to verify interrater reliability; however, some metrics such as perceived racial and ethnic representation remain subjective. Our search terms only included English-language pins about the 4 most common urological cancers. Pins in other languages and those about less common urological malignancies (eg, penile cancer) or benign conditions were not included; these are important areas for further study. Moreover, further research is warranted to understand why some pins received more engagement than others. Currently, we are unable to assess potential associations, that is, the specific country of origin that the pins are from and the type of urological cancers reported. Our results, nevertheless, provide an important and comprehensive snapshot into the type and quality of information on this widely used network.

### Conclusions

In summary, there is a vast array of urological oncology information available on Pinterest, but most of it is of moderate to very poor quality. The importance of addressing and improving eHealth literacy is taking the forefront as the number of individuals using web-based networks increases. The creation of patient-centric information within organizations, which addresses the perspectives and needs of the patients and caregivers, is fundamental [15]. Medical providers can look for credible users on Pinterest to provide higher-quality content. Our study emphasizes the need for collaborative, expert-curated content addressing urological cancers on social media websites like Pinterest.

## Abbreviations

PEMAT: Patient Education Materials Assessment Tool
